# A Medical Causerie

**Published:** 1915-03-06

**Authors:** 


### A MEDICAL CAUSERIE.

The latest figures published by the Panel Committee give an interesting indication of the measure in which the Insurance Act is being worked by the profession in London. The numbers show that 1,449 practitioners in general practice are registered as "panel doctors," and that there are 103 institutions where there are medical officers who treat insured persons. A large proportion of these institutions are hospitals where the nurses and other members of the resident staff come under the operation of the Act and are treated, when necessary, by the house physician or house surgeon. In reference to the practitioners in general practice some deduction must be made for those whose names are on the panel in two or more insurance areas, so that, broadly, the present total shows little increase on the numbers registered some two years ago. The great majority of London practitioners, therefore, are not on the panel, though it must be remembered that a very large percentage of these are not in general practice, and are therefore, for all practical purposes, outside the claim of the Act. The question of the supply of medicines is causing trouble in various parts of the country, and while in view of greater events it is just now pushed into the background, much is likely to be heard of it in the future.

One of the many points at which the war has touched professional interests is, curiously enough, the rights of the "conscientious objector" to vaccination. In ordinary circumstances the father has the legal custody of his child, and the right to object is vested in him. But in his absence the mother succeeds both to his duties and his privileges, and among the latter she may claim the position of a "conscientious objector." There will hardly be question of the justice of this arrangement, but it will be interesting to note whether it makes any appreciable difference in the figures.

It is pleasing to see the increasing support which is being afforded to the Belgian Doctors' and Pharmacists' Relief Fund. The total now reaches some £7,000, and there are in addition Scottish, Irish, and some other local collections. In his speeches in various parts of the country Professor C. Jacobs has made very earnest appeals on behalf of his suffering colleagues. Of the 4,800 doctors in Belgium at least 1,000 are absolutely poverty-stricken, and very many more have suffered severely from the war. The honorary treasurer of the fund is Dr. H. A. Des Vœux, 14 Buckingham Gate, S.W., and gifts of instruments will be received and acknowledged by the Master of the Apothecaries' Company.

Among those whom the present emergency has recalled to active work appears the well-known name of Mr. Mayo Robson, who holds the rank of major and is to go out as consulting surgeon to the Emergency Cases Hospital in France. The hospital is to treat urgent surgical cases among the French wounded, and will be an intermediate station between the firing-line and the base hospitals.

There is much regret that Sir Henry Miers has resigned his position as Principal of the University of London. He has been appointed Vice-Chancellor of the University of Manchester, and the event gives rise to surmises as to how it comes about that the Scottish and provincial universities every now and then manage to tempt some prominent academic figure away from the Metropolitan university. There have recently been two or three instances of movement in the opposite direction, but they are all to appointments hardly of the first order. The vacancies thus filled have been duly advertised, but so much cannot be said of the positions occupied by all the new professors, teachers, and readers lately recognised by the Senate of London University. Apparently it is sufficient for a teacher to be put on the list of one of the medical schools to entitle him to recognition as an academic instructor. This may be all very well when an appointment has been gained after open competition, but methods strictly domestic and private scarcely justify such ready confidence. Why should the university authorities not insist on the advertisement of all appointments where the holders may wish to become recognised teachers?

The Balneological Section of the Royal Society of Medicine is looking ahead, and recognises that the future even of therapeutic measures is likely to be modified by the European war. The Section is to discuss a paper on "The Teutonic Health Resorts and Their Substitutes," to be read by Dr. Leonard Williams. This is certainly a matter for congratulation, for it is inconceivable that English invalids or quasi-invalids will wish to go to Germany for many years, or that they will be welcomed there. Hence the necessity of preparation for some new departure. If health "made in Germany" is no longer available, some other source of supply must be discovered. Other lecture announcements of interest are an intimation that on March 11 Sir Rickman Godlee will speak at the Royal Institution on "Back to Lister," and that Sir Douglas Powell will give a clinical lecture, entitled "A Case of Congenital Heart Disease," at 3 P.M., on March 5, at the Middlesex Hospital.

The advice recently given in The Hospital has not been in vain, and one of the hospitals has courageously advertised for volunteer assistance in the medical and surgical care of its patients. There is every reason to expect that this sensible policy will lead to satisfactory results.

The chronic difficulty in London of obtaining a sufficient number of subjects for the teaching of anatomy and of operative surgery seems recently to have become somewhat acute. The teachers in the various schools have been in conference, and have decided to submit the position to the Home Secretary, though it is not easy to see how this much solicited official can help. There may, however, be methods which can hardly be discussed in public, and certainly the question is a very important one. The efficient teaching of anatomy and operative surgery is so essential that a solution must be found.

